# Supplementary figures and images for: Optical Absorption in N-Dimensional Colloidal Quantum Dot Arrays: Influence of Stoichiometry and Applications in Intermediate Band Solar Cells
Source: Nanomaterials (Basel). 2022 Sep 27;12(19):3387. doi: 10.3390/nano12193387 (PMC9565355; doi:10.3390/nano12193387)

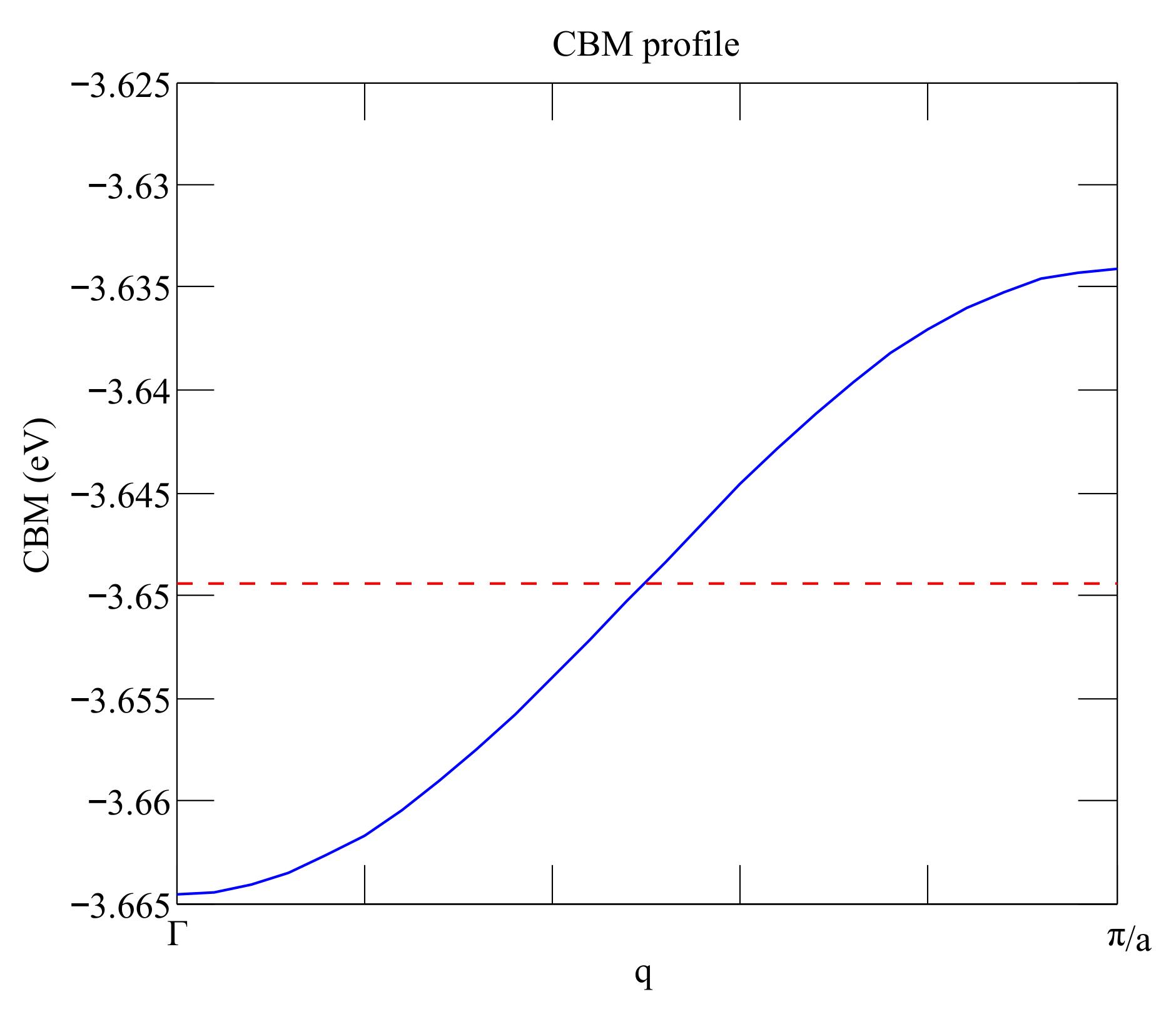

Supplement: Supplementary file 1 [file nanomaterials-12-03387-s001.zip › CBM-profile-SuppInfo.jpg]

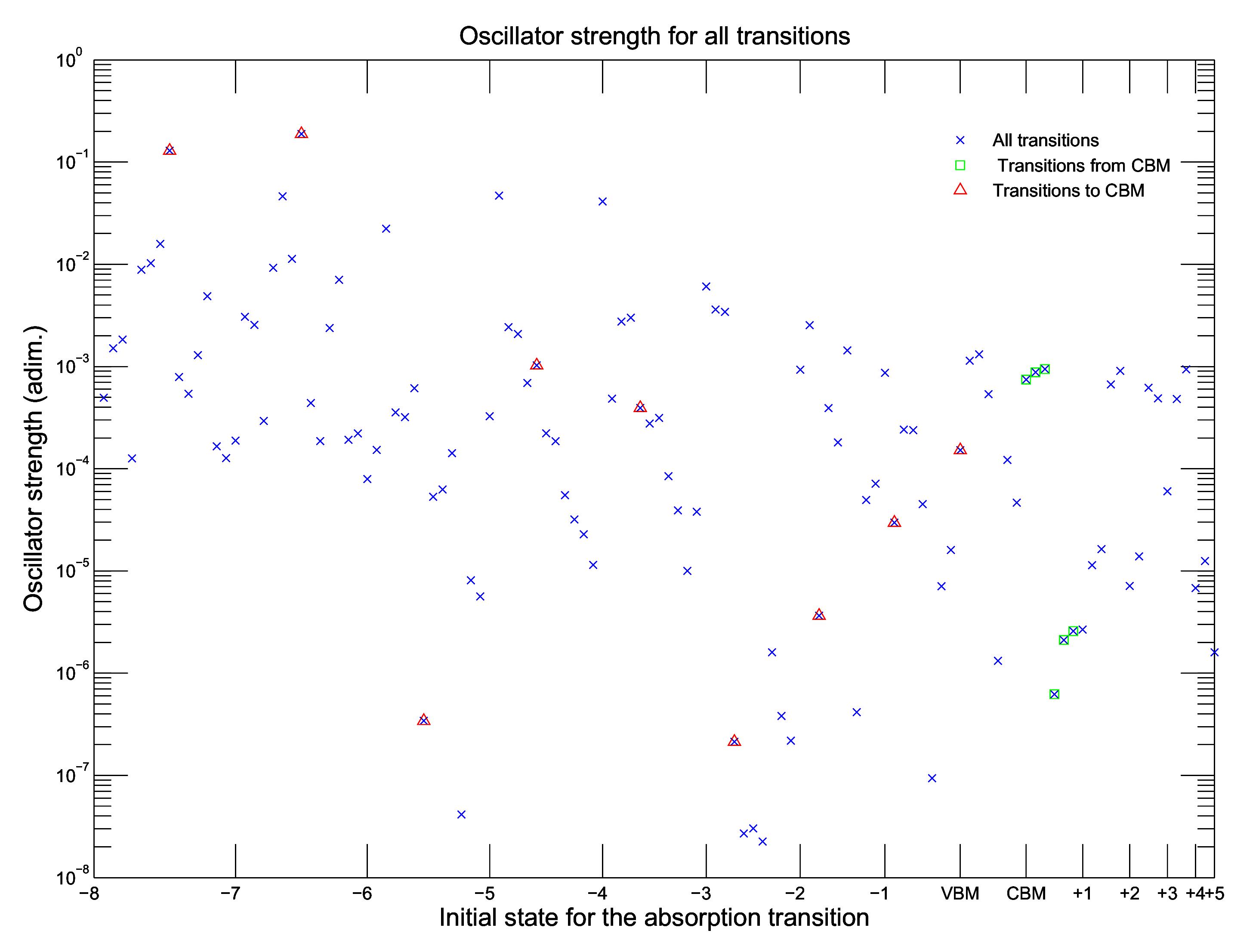

Supplement: Supplementary file 1 [file nanomaterials-12-03387-s001.zip › OS-all-SuppInfo.jpg]

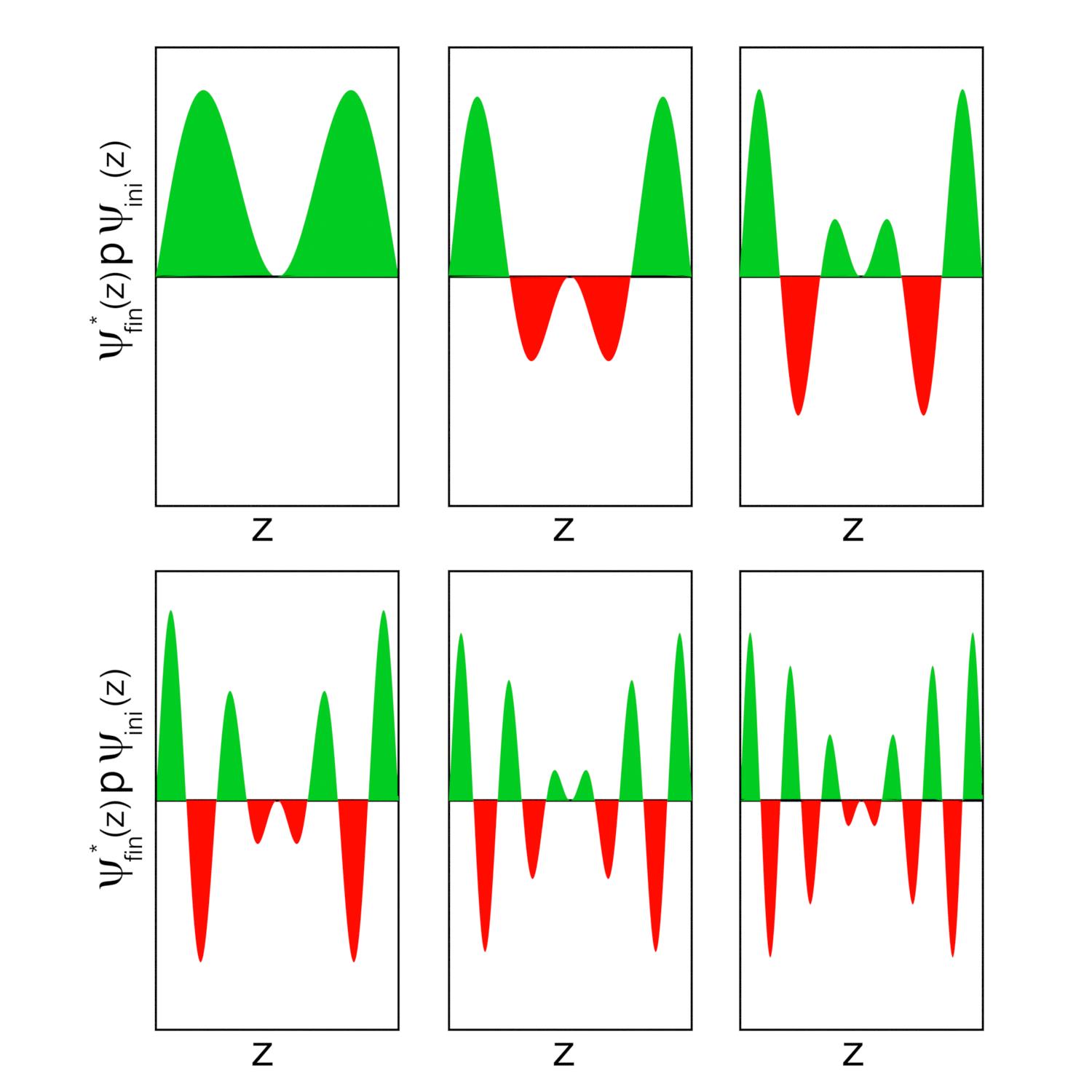

Supplement: Supplementary file 1 [file nanomaterials-12-03387-s001.zip › Oscillations-SuppInfo.jpg]

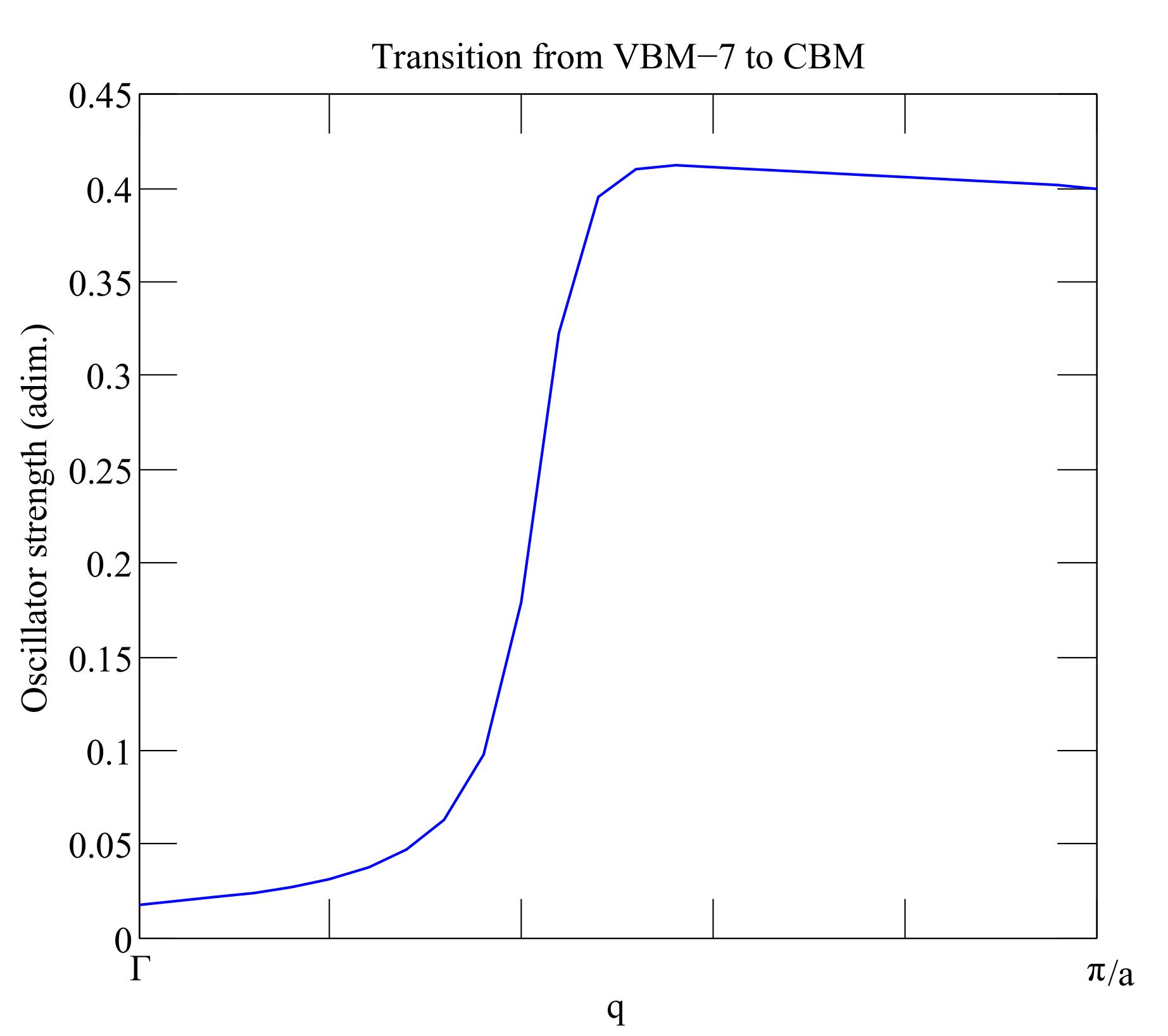

Supplement: Supplementary file 1 [file nanomaterials-12-03387-s001.zip › VBM-7-to-CBM-SuppInfo.jpg]

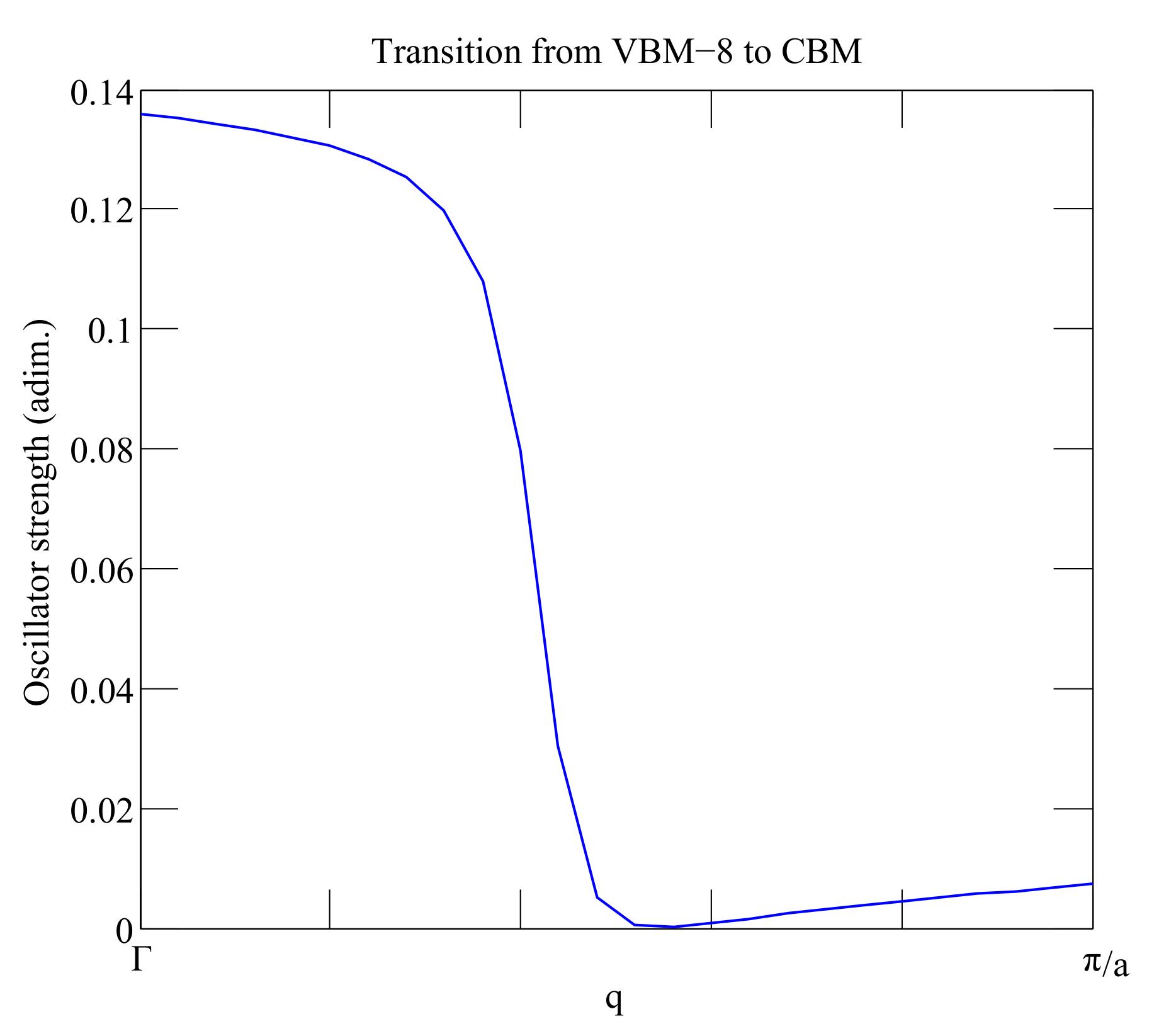

Supplement: Supplementary file 1 [file nanomaterials-12-03387-s001.zip › VBM-8-to-CBM-SuppInfo.jpg]
